# Suggested guidelines for the diagnosis and management of urea cycle disorders

**DOI:** 10.1186/1750-1172-7-32

**Published:** 2012-05-29

**Authors:** Johannes Häberle, Nathalie Boddaert, Alberto Burlina, Anupam Chakrapani, Marjorie Dixon, Martina Huemer, Daniela Karall, Diego Martinelli, Pablo Sanjurjo Crespo, René Santer, Aude Servais, Vassili Valayannopoulos, Martin Lindner, Vicente Rubio, Carlo Dionisi-Vici

**Affiliations:** 1 University Children’s Hospital Zurich and Children’s Research Centre, Zurich, 8032, Switzerland; 2 Radiologie Hopital Necker, Service Radiologie Pediatrique, 149 Rue De Sevres, Paris 15, 75015, France; 3 Department of Pediatrics, Division of Inborn Metabolic Disease, University Hospital Padua, Via Giustiniani 3, Padova, 35128, Italy; 4 Birmingham Children’s Hospital NHS Foundation Trust, Steelhouse Lane, Birmingham, B4 6NH, United Kingdom; 5 Dietetic Department, Great Ormond Street Hospital for Children, NHS Foundation Trust, London, WC1N 3JH, United Kingdom; 6 Kinderabteilung, LKH Bregenz, Carl-Pedenz-Strasse 2, Bregenz, A-6900, Austria; 7 University Children’s Hospital, Medical University Innsbruck, Anichstrasse 35, Innsbruck, 6020, Austria; 8 Division of Metabolism, Bambino Gesù Children’s Hospital, IRCCS, Piazza S. Onofrio 4, Rome, I-00165, Italy; 9 Division of Pediatric Metabolism, Cruces Children Hospital, Baracaldo, 48903, Spain; 10 Universitätsklinikum Hamburg Eppendorf, Klinik für Kinder- und Jugendmedizin, Martinistr. 52, Hamburg, 20246, Germany; 11 Service de Néphrologie et maladies métaboliques adulte Hôpital Necker 149, rue de Sèvres, Paris, 75015, France; 12 Reference Center for Inherited Metabolic Disorders (MaMEA), Hopital Necker-Enfants Malades, 149 Rue de Sevres, Paris, 75015, France; 13 University Children’s Hospital, Im Neuenheimer Feld 430, Heidelberg, 69120, Germany; 14 Instituto de Biomedicina de Valencia del Consejo Superior de Investigaciones Científicas (IBV-CSIC) and Centro de Investigación Biomédica en Red para Enfermedades Raras (CIBERER), C/ Jaume Roig 11, Valencia, 46010, Spain

**Keywords:** Urea cycle disorders, UCD, Hyperammonemia, N-acetylglutamate synthase, Carbamoylphosphate synthetase 1, Ornithine transcarbamylase, Ornithine carbamoyl transferase, Argininosuccinate synthetase, Argininosuccinate lyase, Arginase 1, Hyperornithinemia-hyperammonemia-homocitrullinuria syndrome

## Abstract

Urea cycle disorders (UCDs) are inborn errors of ammonia detoxification/arginine synthesis due to defects affecting the catalysts of the Krebs-Henseleit cycle (five core enzymes, one activating enzyme and one mitochondrial ornithine/citrulline antiporter) with an estimated incidence of 1:8.000. Patients present with hyperammonemia either shortly after birth (~50%) or, later at any age, leading to death or to severe neurological handicap in many survivors. Despite the existence of effective therapy with alternative pathway therapy and liver transplantation, outcomes remain poor. This may be related to underrecognition and delayed diagnosis due to the nonspecific clinical presentation and insufficient awareness of health care professionals because of disease rarity. These guidelines aim at providing a trans-European consensus to: guide practitioners, set standards of care and help awareness campaigns. To achieve these goals, the guidelines were developed using a Delphi methodology, by having professionals on UCDs across seven European countries to gather all the existing evidence, score it according to the SIGN evidence level system and draw a series of statements supported by an associated level of evidence. The guidelines were revised by external specialist consultants, unrelated authorities in the field of UCDs and practicing pediatricians in training. Although the evidence degree did hardly ever exceed level C (evidence from non-analytical studies like case reports and series), it was sufficient to guide practice on both acute and chronic presentations, address diagnosis, management, monitoring, outcomes, and psychosocial and ethical issues. Also, it identified knowledge voids that must be filled by future research. We believe these guidelines will help to: harmonise practice, set common standards and spread good practices with a positive impact on the outcomes of UCD patients.

## Introduction

**Figure 1:**
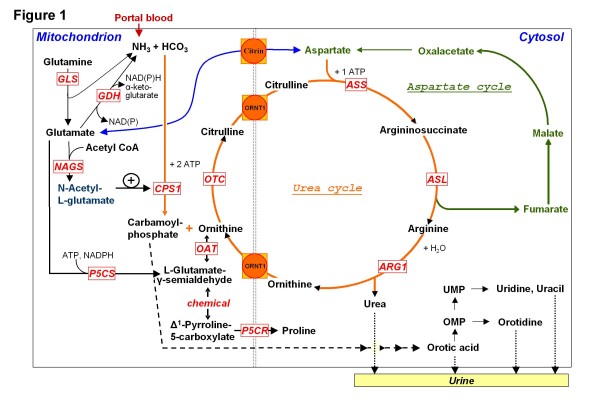
**The urea cycle and associated pathways.** Non-standard abbreviations include: GDH, glutamate dehydrogenase; GLS, glutaminase; NAD(P), nicotinamide adenine dinucleotide (phosphate); OAT, ornithine aminotransferase; OMP, orotidine monophosphate; P5CR, pyrroline-5-carboxylate reductase; P5CS, Δ^1-^pyrroline-5-carboxylate synthetase; UMP, uridine monophosphate.

Urea cycle disorders (UCDs) are inborn errors of nitrogen detoxification/arginine synthesis due to defects in the urea cycle enzymes (Figure 1), carbamoylphosphate synthetase 1 (CPS1), ornithine transcarbamylase (OTC), argininosuccinate synthetase (ASS), argininosuccinate lyase (ASL) and arginase 1 (ARG1), leading to respective deficiencies (abbreviated CPS1D, OTCD, ASSD, ASLD and ARG1D; corresponding MIM numbers, #237300, #311250; #215700; #207900; #207800 respectively). They also encompass deficiencies of N-acetylglutamate synthase (NAGS) (MIM #237310), associated with lack of the N-acetylglutamate (NAG) essential activator of CPS1 and of the mitochondrial ornithine/citrulline antiporter (ORNT1), causing the hyperornithinemia-hyperammonemia-homocitrullinuria (HHH) syndrome (MIM #238970). The prevalence of these disorders may exceed the current estimates (1:8,000-1:44,000 births [1-3], for all UCDs jointly) because of unreliable newborn screening and underdiagnosis of fatal cases. Clinical features are typical in complete deficiencies, which present with hyperammonemic coma a few days after birth with ~50% mortality [4-7], whereas the survivors experience severe developmental delay and recurrent hyperammonemic crises [4-7]. Even in partial deficiencies, which have more variable clinical presentations and later onset (any age), there is increased risk of premature death [5,8]. The duration and severity of hyperammonemia strongly correlates with brain damage [6,9,10]; prompt diagnosis and treatment of UCD is essential in order to optimise the outcome. [11]. However, the rarity of UCDs prevents single centres or even countries to have all the expertise for evidence-based management. Therefore, we have developed consensus guidelines based on the highest available level of evidence, by pooling all the published evidence and experience of leading centres from several European countries, to help standardise, systematise and harmonise across Europe the diagnosis, therapy, procedures and management of UCDs. These guidelines, developed with the Delphi methodology are intended to be used by metabolic specialists, pediatricians, dietitians, neonatologists, intensive care specialists, adult physicians, neurologists, nurses, psychologists and pharmacists involved in the care of UCD patients. Excluded from these guidelines because of insufficient European experience, or of tangential relationship with UCDs are: citrin deficiency (citrullinemia type 2, MIM #605814 and #603471), lysinuric protein intolerance (LPI, MIM #222700), deficiencies of pyrroline 5-carboxylate synthetase (MIM #610652) and ornithine aminotransferase deficiency (OAT, MIM #258870), despite the fact that they may cause hyperammonemia.

## Methodology and objectives

### Guidelines development

Development of these guidelines spanned the time period, October 2008 until August 2011 and involved one preliminary meeting and four working meetings of the guideline development group (GDG), formed by pediatric metabolic specialists (S. Baumgartner [Innsbruck, retired after the first meeting], AB, AC, CDV, S. Grünewald, [London, retired after the first meeting], JH [chairman], DK, ML [secretary], DM, PS, VV), a medical biochemist (VR), a psychologist (MH), a specialist metabolic dietitian (MD), a metabolic specialist caring for adult patients (AS) and a neuroradiologist (NB). Each meeting was supervised by a moderator (P. Burgard, Heidelberg [first meeting] and RS) who oversaw the discussion but did not contribute to the content. In the initial working meeting the GDG was trained on standardising literature evaluation and working groups focusing on specific topics were formed. Thereafter GDG members discussed and performed systematic literature review and drafted the guidelines. These drafts were further reviewed by external specialists on intensive care (L. Dupic, Paris), genetics (A. Gal, Hamburg), child neurology (A. Garcia-Cazorla, Barcelona), nephrology (S. Picca, Rome), liver transplantation (J. de Ville de Goyet, Rome), epidemiology (A. Tozzi, Rome) and ethics (C. Rehmann-Sutter, Basel) and a patient group representative (S. Hannigan, London). After further recommendations/comments by three highly renowned external reviewers (C. Bachmann, Bottmingen; J.V. Leonard, Oxford and H. Ogier, Paris), the final version of the guidelines was written and its applicability pilot-tested by non-specialist pediatricians in training, with subsequent review and revision by the GDG. The guidelines will be sent for endorsement to all European societies for inherited metabolic diseases.

### Systematic literature review and evidence grading

The guidelines evidence base was collected according to the Scottish Intercollegiate Guideline Network (SIGN, http://www.sign.ac.uk). Systematic literature review encompassing from each disease description until early 2011 was carried out using mainly Medline, Embase, the Cochrane Library, MedLink, and Orphanet. Searches also included websites of societies and parents groups for inborn errors. Relevant papers were evaluated by at least two GDG members before considering conclusions as evidence.

Evidence levels were classified in accordance with the SIGN methodology:

"Evidence level & criteria"

1^++^ High quality meta-analyses, systematic reviews of randomized control trials (RCTs), or RCTs with a very low risk of bias.

1^+^ Well conducted meta-analyses, systematic reviews of RCTs, or RCTs with a low risk of bias.

1^-^ Meta-analyses, systematic reviews or RCTs, or RCTs with a high risk of bias.

2^++^ High quality systematic reviews of case–control or cohort studies or high quality case–control or cohort studies with a very low risk of confounding bias, or chance and a high probability that the relationship is causal.

2^+^ Well conducted case–control or cohort studies with a low risk of confounding, bias, or chance and a moderate probability that the relationship is causal.

2^-^ Case–control or cohort studies with a high risk of confounding, bias, or chance and a significant risk that the relationship is not causal.

3 Non-analytic studies, e.g. case reports, case series.

4 Expert opinion.

Recommendations given in the guidelines are graded depending on their level of evidence:

"Grade of recommendation & criteria"

A If level 1 evidence was found (not the case).

B If level 2 evidence was found.

C If level 3 evidence was found (mainly non-analytical studies such as case reports and case series).

D If level 4 evidence was found (mainly expert opinion).

### Disclaimer

These guidelines aim at helping decision making in UCD patient care. Although based on the best available evidence, the recommendations given often reflect only expert opinion and are thus not meant to be rigidly implemented. Furthermore, although as exhaustive as possible, these guidelines cannot include all possible methods of diagnostic work-up and care and may therefore fail to mention some acceptable and established procedures. Guidelines cannot guarantee satisfactory diagnosis and outcome in every patient. Although helping optimise the care of individual patients and assist decision-making by basing clinical practice on the existing scientific and medical knowledge, they should not substitute well-informed, prudent clinical practice.

## Diagnosis

### The clinical picture

The clinical manifestations of UCDs (Table 1) can occur at any age [12-16], with hyperammonemic crises being frequently triggered by catabolic events, protein overload or certain drugs. Most symptoms are neurological but nonspecific. A UCD should be immediately suspected in neonates if there are any neurological symptoms or at any age if there is an acute encephalopathy. Hepatic-gastrointestinal and psychiatric nonspecific manifestations (Table 1) are second in frequency. Only the hair shaft abnormalities with hair fragility (trichorrhexis nodosa) found mainly in ASLD [12,17-19] and the progressive spastic diplegia beginning in childhood (or later) in ARG1D and the HHH syndrome, frequently without hyperammonemic episodes [20-22], are specific manifestations of this group of diseases. Symptoms can be subtle, particularly after the neonatal period, and in some patients symptomatic episodes can resolve with nonspecific interventions. Women can first manifest a UCD as acute unexplained neurological symptoms in the postpartum period (reported for CPS1D, OTCD, and ASSD [23-25]). Variability in disease severity is characteristic for OTCD heterozygous females (due to lyonization) [11,26], but is also found in all UCDs, being mainly attributable to differences in the severity of the genetic change [27-30]. However, the same genetic defect can yield both mild and severe presentations even in different members of the same family (reported for OTCD and for one CPS1D family) [31-33]. Acute liver failure has been reported as the presenting sign in patients with OTCD, ASSD and HHH syndrome [34-39]. Although rare, a number of other presentations have been reported in UCDs, including stroke-like episodes (metabolic strokes) [10,40-44] that may resolve with treatment, chorea [45], cerebral palsy without hyperammonemia or cerebral edema [46,47], episodic transient or protracted cortical visual losses [48,49], dermatitis (most probably because of treatment-related malnutrition) [50,51], autism-like symptoms [52,53], behavioural problems during childhood [53] and in postpuberal patients and other episodic psychiatric symptoms that may be the only manifestation [54].

**Table 1:** Clinical signs and symptoms of acute and chronic presentations of UCDs, and triggering factors for hyperammonemia in UCD patients

**Acute presentation**	**Chronic presentation**
**· Altered level of consciousness (from somnolence and lethargy to coma) mimicking encephalitis or drug intoxication**	**· Confusion, lethargy, dizziness**
	**· Migraine-like headaches, tremor, ataxia, dysarthria**
**· Acute encephalopathy (see below)**	**·** Asterixis (in adults)
**· Seizures (generally not isolated but along with an altered level of consciousness)**	**· Learning disabilities, neurodevelopmental delay, mental retardation**
**·** Ataxia (generally associated with altered consciousness level)	**·** Chorea, cerebral palsy
**·** Stroke-like episodes	**·** Protracted cortical visual loss
**·** Transient visual loss	**·** Progressive spastic diplegia or quadriplegia (described in ARG1D and HHH syndrome)
**· Vomiting and progressive poor appetite**	**· Protein aversion, self-selected low-protein diet**
**·** Liver failure	**· Abdominal pain, vomiting**
**· Multiorgan failure**	**· Failure to thrive**
**· Peripheral circulatory failure**	**· Hepatomegaly, elevated liver enzymes**
**·** “Post-partum psychosis”	**· Psychiatric symptoms: hyperactivity, mood alteration, behavioural changes, aggressiveness**
**· Psychiatric symptoms (hallucinations, paranoia, mania, emotional or personality changes)**	**·** Self-injurious behaviour
	**·***Autism-like symptoms*
** In neonates: **	**· Fragile hair (typical for ASLD)**
**· sepsis-like picture, temperature instability** **· respiratory distress, hyperventilation**	**·***Dermatitis*
	**·** Specific neuropsychological phenotype in heterozygous OTC females
	**· Episodic character of signs and symptoms**
**Potential triggers of hyperammonemic crises in UCD patients**	
**·** Infections	
**·** Fever	
**·** Vomiting	
**·** Gastrointestinal or internal bleeding	
**·** Decreased energy or protein intake (e.g. fasting pre surgery, major weight loss in neonates)	
**·** Catabolism and involution of the uterus during the postpartum period (mostly OTC females)	
**·** Chemotherapy, high-dose glucocorticoids	
**·** Prolonged or intense physical exercise	
**·** Surgery under general anesthesia	
**·** Unusual protein load (e.g. a barbecue, parenteral nutrition)	
**·** Drugs: Mainly **valproate and L-asparaginase/pegaspargase.** Topiramate, carbamazepine, phenobarbitone, phenytoine, primidone, furosemide, hydrochlorothiazide and salicylates have also been associated with hyperammonemic decompensation.	

Typical and uncommon signs and symptoms are highlighted in bold- and normal-type, respectively, whereas italic type marks signs and symptoms reported in single patients. *Grade of recommendation, D*.

A careful medical and family history is mandatory and should include questions about unexplained neonatal deaths, neurological or psychiatric disorders in the family, consanguinity (frequent in all UCDs except in OTCD, which is X-linked), evidence of protein avoidance in patient and family members and drug intake by the patient.

#### Statement #1. Grade of recommendation: C

UCDs may present with acute or chronic presentations at any age and are often triggered by catabolic events, protein load or some drugs. In many cases a precipitating factor cannot be identified. Clinical signs and symptoms are nonspecific and commonly neurological, gastrointestinal or psychiatric. It is essential that healthcare professionals have an awareness of these diseases. Key questions should be asked and a detailed family history with pedigree is mandatory.

#### Statement #2. Grade of recommendation: D

UCDs must be included in the differential diagnosis of acute unexplained encephalopathy or acute psychiatric illness at any age, which must prompt plasma ammonia determination.

### Laboratory findings

*Hyperammonemia,* a nonspecific marker of inadequate nitrogen detoxification [55], is the hallmark for most UCDs. The absence of hyperammonemia in symptomatic newborn patients (but not in older patients) renders a UCD highly unlikely. Rapid ammonia measurement in an emergency setting is crucial since patient outcome correlates with the duration and peak level of hyperammonemia [4,6,56]. Respiratory alkalosis in a newborn should prompt immediate ammonia measurement because it is present initially in 50% of acute UCDs [5]. Otherwise the acid–base status is of limited use [57].

#### Statement #3. Grade of recommendation: C

Ammonia should be determined in an emergency setting with results available in 30 minutes.

#### Statement #4. Grade of recommendation: D

Ammonia should be measured in patients of any age presenting 1) an unexplained change in consciousness; 2) unusual or unexplained neurological illness; 3) liver failure; 4) suspected intoxication.

If hyperammonemia is confirmed, determination of plasma amino acids, blood or plasma acylcarnitines, urinary organic acids and orotic acid should be urgently requested together with basic laboratory investigations, not waiting for the results (which should be obtained in <24 h) for treating the patient. When taking samples after recovery from an acute episode, plasma amino acid levels and/or urinary orotic acid (measured with a specific method e.g. high performance liquid chromatography) can be particularly helpful for diagnosis. In patients with fatal outcome, procurement of anticoagulated blood for DNA isolation and storage of frozen aliquots of all samples obtained of plasma, serum, urine and cerebrospinal fluid (CSF) is recommended [16,58].

#### Statement #5. Grade of recommendation: D

If ammonia is found elevated, further metabolic investigations should be immediately carried out without delaying specific treatment.

## Differential diagnosis

The most common misdiagnosis of early onset UCD patients is neonatal sepsis. A number of conditions that increase ammonia production and/or secondarily decrease ammonia detoxification can cause hyperammonemia and mimic a UCD [16,59-63]. Thus, * neonatal hyperammonemia * can be due to UCDs, to other inborn errors that cause secondary hyperammonemia*,* to liver failure or to congenital infection. Premature infants can have transient hyperammonemia, a condition which is characterised by a normal blood glutamine level [64] and which is possibly due to ductus venosus shunting of portal blood [65-67]. * Late-onset hyperammonemia * can be triggered by most conditions that can also cause neonatal hyperammonemia, by chronic liver failure, exogenous intoxications (e.g. amanita phalloides), drugs (e.g. valproic acid), porto-caval shunt and Reye syndrome, by conditions that vastly increase either direct ammonia production (e.g. asparaginase treatment, urease-positive bacteria overgrowth or genito-urinary infection) or protein catabolism (e.g. myeloma, chemotherapy, steroid therapy, trauma, gastrointestinal hemorrhage) and when there is excessive nitrogen supply (reported in total parenteral nutrition or after glycine-solution irrigations in transurethral prostate resection) [5,17,68-72]. Table 2 lists errors of metabolism leading to hyperammonemia, guiding bedside differentiation.

**Table 2:** Bedside differential diagnosis of inborn errors of metabolism presenting with hyperammonemia

**Parameter**	**Condition**				
	**UCDs**	**Organic acidurias**	**β-Oxidation defects**	**Hyperinsulinism- hyperammonemia syndrome**	**Pyruvate carboxylase deficiency**^g^
**Acidosis**	+**/–**	+ ^**e**^	+**/–**	**–**	+
**Ketonuria**^**a**^	**–**	+	**–**	**–**	++
**Hypoglycemia**^**b**^	**–**	+**/–**	+	+	+
**↑ Lactic acid**^**c**^	**–**	+	+**/–**	**–**	+
**↑ AST & ALT**	**(**+**)**^**d**^	**–**	+	**–**	+**/–**
**↑ CPK**	**–**	**–**	+	**–**	**–**
**↑ Uric acid**	**–**	+	+	**–**	**–**
**↓ WBC/RBC/Plt**	**–**	+	**–**	**–**	**–**
**Weight loss**	**–**	+^**f**^	**–**	**–**	+

In addition to the conditions indicated in the table, mitochondrial oxidative phosphorylation defects, citrin deficiency, lysinuric protein intolerance or ornithine aminotransferase deficiency can also cause hyperammonemia.

*Grade of recommendation, D*.

^a^ In neonates ketonuria (++ or +++) suggests organic aciduria.

^b^ Hypoglycemia and hyperammonemia (“pseudo-Reye”) can be predominant manifestations of the organic aciduria due to 3-hydroxy-3-methylglutaryl-CoA lyase deficiency.

^c^ Blood lactate >6mmol/L, since lower high lactate levels (2-6mM) may be due to violent crying or to extensive muscle activity.

^d^ AST & ALT elevations can be found but are not constant in UCDs.

^e^ Can be absent in neonates.

^f^ Occurrence only in neonates.

^g^ Only type B is associated with hyperammonemia but not types A and C.

### *Statement #6. Grade of recommendation: C*

In newborns with clinical distress where sepsis is suspected, hyperammonemia must always form part of the initial differential diagnosis.

**Figure 2:**
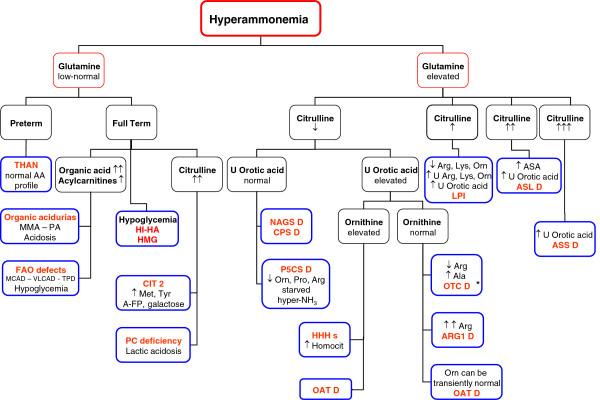
**Diagnostic algorithm for neonatal hyperammonemia.** Unless indicated, plasma is used for the analytical determinations. Non-standard abbreviations include: A-FP, α fetoprotein; CIT 2, citrullinemia type 2; CPSD, CPS1 deficiency; HI-HA, hyperinsulinism-hyperammonemia syndrome; HMG, 3-hydroxy-3-methylglutaryl-CoA lyase deficiency; LPI, lysinuric protein intolerance; OATD, ornithine aminotransferase deficiency; PA, propionic acidemia; PC, pyruvate carboxylase; P5CSD, Δ^1-^pyrroline-5-carboxylate deficiency; THAN, transient hyperammonemia of the newborn; TPD, trifunctional protein deficiency; U, urine. *Grade of recommendation, D.* * In some patients with late-onset OTCD, plasma citrulline levels are in the lower part of the normal range.

Standard clinical and analytical procedures generally differentiate between hyperammonemia due to inborn errors and that due to other conditions such as liver failure [1,16,73-75]. The algorithm given in Figure 2 guides the identification of the specific defect when the hyperammonemia is due to an inborn error. ARG1D and ASLD can be identified, respectively, by the high plasma arginine or the high plasma/urinary argininosuccinate (ASA) level. The finding of high plasma citrulline in the absence of ASA is highly suggestive of ASSD. The combination of hyperammonemia with low plasma citrulline and arginine is diagnostic of OTCD when orotic acid is increased in the urine, whereas it strongly suggests CPS1D or NAGS deficiency (NAGSD) when urinary orotic acid is low. The finding of high plasma ornithine and hyperammonemia, (these two traits can also be found in OAT deficiency) with high urinary homocitrulline is characteristic of the HHH syndrome. When the metabolite pattern is not clear-cut, activity assays of urea cycle enzymes in liver (all urea cycle enzymes), red blood cells (ASL and ARG1; still very useful in ARG1D [76]), intestinal mucosa (CPS1, OTC) or fibroblasts (ASS, ASL, HHH) can clarify diagnosis, although enzyme assays have generally been replaced by genetic testing. Enzyme analysis is now mainly reserved for the minority of cases in whom genetic analysis fails to identify a specific UCD (see below).

### *Statement #7. Grade of recommendation: D*

Genetic testing is the method of first choice to confirm the diagnosis. Liver tissue, intestinal mucosa, erythrocytes and fibroblasts can be used for enzyme activity assays in UCDs if genetic testing does not identify a specific UCD, or if it is not available. In deceased patients with a suspicion of UCD, fibroblasts and/or liver tissue should be preserved frozen.

## Molecular genetic analysis

Except for OTCD, which is transmitted in the X-chromosome, UCDs exhibit autosomal recessive inheritance [12-16]. Mutations in the corresponding genes (homonymous with the enzymes) have been identified in patients of all UCDs (see http://www.ncbi.nlm.nih.gov/sites/entrez?db=omim) including citrullinemia type 2 (*SLC25A15* gene encoding citrin) and the HHH syndrome (*SLC25A13* gene). Mutation detection has at least ~80% sensitivity [77] and permits carrier identification, prenatal diagnosis, facilitating pedigree analysis, genetic counselling and in some cases genotype-phenotype correlations [15,27,78], conceivably opening the way to future therapies (e.g. nonsense read-through approaches). DNA, generally from blood, is used, although the large number of *CPS1* exons renders preferable the utilization of RNA from cultured fibroblasts for CPS1D studies. For other UCDs RNA analysis (from liver in the case of OTCD) is only carried out when DNA analysis is negative [79-81]. Prognostic judgements on the disease-causing nature of missense mutations (the most frequent ones) and of some splice-site mutations are difficult if not backed by in vitro expression studies of the mutant protein.

### *Statement #8. Grade of recommendation: C*

Mutation analysis is the method of choice for definitive diagnosis of UCDs, to help with genetic counselling and in some instances indicate the prognosis.

### *Statement #9. Grade of recommendation: D*

Mutation analysis has some pitfalls and limitations, including the difficulty in establishing the pathogenic potential of a missense mutation. *In vitro* protein expression studies and in silico analyses based on sequence conservation and protein structure can help infer pathogenic potential but are not part of routine clinical management.

## Prenatal testing

Prenatal investigations in UCDs are available in many countries and may enable pregnancy termination of affected foetuses. These may also be indicated in milder UCDs or for NAGSD (which has substitutive therapy) for psychological reasons and to prepare for perinatal management [82-84]. Among the techniques that can be used (Table 3), mutation- or disease allele-tracking using chorionic villus samples, amniotic fluid cells or cultures thereof [85,86] is the method of choice since it gives rapid and clear-cut results relatively early on, with little fetal risk. Amniotic fluid citrulline and ASA determinations are also suitable for respective ASSD and ASLD prenatal diagnosis [86-88].

**Table 3:** Prenatal testing of UCDs: Recommended analyses and sample requirements

**Disorder**	**Recommended tests**
NAGSD	Mutation analysis using DNA from CVS or AFC^a^
CPS1D	**Mutation analysis using DNA from CVS or AFC**
	Enzyme assay in late fetal liver biopsy^b^
OTCD	**Mutation analysis using DNA from CVS or AFC**^**c**^
	Enzyme assay in late fetal liver biopsy^b,d^
ASSD	**Mutation analysis using DNA from CVS or AFC**
	**Citrulline in amniotic fluid**
	Enzyme assay in intact or cultured CVS or in cultured AFC
ASLD	**Mutation analysis using DNA from CVS or AFC**
	**Argininosuccinate and its anhydrides in amniotic fluid**
	Enzyme assay in intact or cultured CVS or cultured AFC
ARG1D	**Mutation analysis using DNA from CVS**
	**Enzyme assay in fetal blood erythrocytes (mid-gestation sampling)**
HHH syndrome	**Mutation analysis using DNA from CVS or AFC**
	Enzyme assay in CVS or cultured AFC

First choices are given in bold-type. CVS, chorionic villus sampling. AFC, amniotic fluid cells. *Grade of recommendation, D*.

^a^ The woman should be informed prior to prenatal testing that in NAGSD the phenotype can be normalized completely with life-long substitutive therapy.

^b^ Very limited experience (single patient report) and test not widely available.

^c^ The presence of one mutation in a female fetus cannot predict the phenotype given the effect of lyonization.

^d^ Informative in males but interpretation not clear in females due to lyonization-caused X-mosaicism.

### *Statement #10. Grade of recommendation: D*

Prenatal testing requires joint careful counselling by clinical geneticists and metabolic specialists.

### *Statement #11. Grade of recommendation: C-D*

Molecular genetic analysis is the preferred prenatal testing method for all UCDs. Investigations of metabolites in amniotic fluid and of enzyme activities in chorionic villi, cultured amniotic cells, fetal liver or fetal erythrocytes can also be used.

## Newborn screening (NBS)

UCD patients manifesting severe neonatal hyperammonemia benefit little from NBS or even from early diagnosis, because of their poor prognosis [89-91] although the family would benefit from knowing the diagnosis. However, NAGSD, CPS1D and OTCD are generally not screened for, given the instability of glutamine and the low specificity and sensitivity for detection of decreases in the citrulline level [92]. The benefits of screening for ASSD, ASLD, and ARG1D, carried out in most US states, Taiwan and Australia by assessing respectively citrulline, ASA and arginine levels in dried blood spots, have not yet been formally evaluated. Although for severe ASSD and ASLD there are few false positives and no false negatives [93-95], ASLD screening was abandoned in Austria because of the high rate of positive newborns probably having partial deficiency but remaining asymptomatic [96]. The sensitivity of NBS for ARG1D and HHH is unknown, since in these diseases arginine and ornithine levels, respectively, may be normal in the first days of life [97]. A further difficulty with HHH is the potential production of ornithine by red cell arginase as the blood spot dries.

### *Statement #12. Grade of recommendation: D*

Newborn screening for NAGSD, CPS1D and OTCD cannot currently be recommended.

### *Statement #13. Grade of recommendation: C*

Newborn screening for ASSD, ASLD, and ARG1D may be considered, but more information on the resulting benefits and problems is needed.

## Management of acute hyperammonemia

### Initial management

Since prognosis is strongly influenced by the duration of coma [6,56] and peak ammonia levels [9,98,99], therapy must not be delayed. Pediatric hospitals should always have first-line medications and consensus-based written protocols on how to proceed. Patients in hyperammonemic crisis should be transferred without delay to a specialist centre after:

1) stopping protein intake

2) starting intravenous (IV) 10% glucose

3) initiation of first-line medications as outlined in Table 4

4) plasma and urine collection for diagnostic purposes without postponing treatment initiation.

**Table 4:** **Levels of hyperammonemia and suggested actions in symptomatic patients *Grade of recommendation, C-D ***

**Ammonia level (μmol/L)**	**Action in undiagnosed patient**	**Action in known UCD patient**	**Comments**
Above upper limit of normal	▪ Stop protein intake	▪ Stop protein intake	▪ Stop protein for 24 h (maximum 48 h)
	▪ Give IV glucose at an appropriate dosage to prevent catabolism (10mg/kg/min in a neonate) ± insulin^a^	▪ Give IV glucose at an appropriate dosage to prevent catabolism (10mg/kg/min in a neonate) ± insulin^a^	▪ Avoid exchange transfusions as they cause catabolism
	▪ Monitor blood ammonia levels every 3 h	▪ Monitor blood ammonia levels every 3 h	▪ Hyperglycemia can be extremely dangerous (hyperosmolarity)
**In addition**			
if >100 and <250 (in neonates, >150 and <250)	▪ Start drug treatment with IV L-arginine and nitrogen scavengers (see Table 5)	▪ Continue drug treatment with L-arginine (plus continue or add L-citrulline for NAGSD, CPS1D or OTCD) and sodium benzoate ± sodium phenylbutyrate/ phenylacetate^b^ (see Table 5), increase dose or give IV	▪ If major hyperglycemia occurs with high lactate (>3mmol/L) reduce glucose infusion rate rather than increase insulin
	▪ Start carbamylglutamate, carnitine, vitamin B12, biotin (see Table 5 and its legend)	▪ Consider nasogastric carbohydrate and lipid emulsions unless the child is vomiting (enables higher energy intake)	▪ Avoid hypotonic solutions
**In addition**			
if 250 to 500	▪ As above	▪ As above, but all drugs per IV	▪ Add sodium and potassium according to the electrolyte results
	▪ Prepare hemo(dia)filtration if significant encephalopathy and/or early high blood ammonia level or very early onset of disease (day 1 or 2)	▪ Prepare hemo(dia)filtration if significant encephalopathy and/or early high blood ammonia level or very early onset of disease (day 1 or 2)	▪ Take into account the sodium intake if sodium benzoate or sodium PBA are used ^c^
	▪ Begin hemo(dia)filtration if no rapid drop of ammonia within 3–6 h	▪ Begin hemo(dia)filtration if no rapid drop of ammonia within 3–6 h	▪ L-arginine not to be given in ARG1D
**In addition**			
if 500 to 1000	▪ As above	▪ As above	▪ Some concerns of sodium benzoate use in organic acidemias
	▪ Start hemo(dia)filtration immediately	▪ Start hemo(dia)filtration as fast as possible	▪ Avoid repetitive drug boluses
**In addition**			
if >1000	▪ Evaluate whether to continue specific treatment or to start palliative care	▪ Evaluate whether to aim at curative treatment or at palliative care	▪ Monitor phosphate levels and supplement early specially with hemodialysis

^a^ Monitor blood glucose after 30 min and subsequently every hour, because some neonates are very sensitive to insulin.

^b^ If available, an IV equimolar solution of sodium benzoate and sodium phenylacetate can be used: 250mg/kg as bolus IV/90-120 min, then 250mg/kg as continuous IV infusion over 24h.

^c^ Sodium content in 1 gram of sodium benzoate or sodium phenylbutyrate, 7 mmol and 5.4 mmol, respectively.

The diagnostic workup of the specific defect and the initial medical treatment should proceed simultaneously.

#### Statement #14. Grade of recommendation: C-D

Early clinical suspicion and prompt diagnosis of hyperammonemia are crucial for a favourable outcome. The start of ammonia detoxification and of measures to reverse catabolism must not be delayed unless a decision for withdrawal of treatment and for palliative care is made.

Before treatment of acute hyperammonemia, the prognosis and expected neurodevelopmental outcome must be considered since it can influence the therapeutic decision. Prognosis is considered very poor if:

1. hyperammonemic coma has lasted more than 3 days

2. intracranial pressure is clearly increased

3. ammonia peaked at >1000 μmol/L [4,56] although the impact of this level on prognosis depends on the duration of hyperammonemia.

#### Statement #15. Grade of recommendation: C

Total duration of coma and peak ammonia levels are the most relevant factors for neurodevelopmental prognosis. More studies are needed to identify other potential contributing factors.

### Drugs and dosages in acute UCD decompensations

The ammonia scavengers sodium benzoate, sodium phenylacetate or sodium phenylbutyrate (PBA) are the mainstay drugs for bypassing the urea cycle, by conjugation of benzoate with glycine to generate hippurate, or of phenylacetate (phenylbutyrate is a precursor of phenylacetate) with glutamine to generate phenylacetylglutamine. These conjugates are excreted in the urine [1,16,73,75,98,100,101]. Arginine and/or citrulline administration aims at maximising ammonia excretion through the urea cycle [12-16], whereas N-carbamylglutamate replaces the CPS1 activator N-acetylglutamate ( [12,16] and see below). The dose ranges given in Table 5 reflect a consensus of this guideline working group and are supported by several publications [1,16,73,75,98,100,101]. Ondansetron (0.15 mg/kg) may be administered to avoid vomiting when boluses of the scavengers are administered [101]. Repeated boluses or very high doses of benzoate, phenylbutyrate/phenylacetate can saturate the scavenger-converting systems and increase the risk of drug accumulation and toxicity [102].

**Table 5:** Consensus guidelines for drug dosages for acute hyperammonemia and acute decompensations of UCDs

**Disorder**	**Sodium benzoate** **(to be given IV in 10% glucose)**	**Sodium PBA/Sodium phenylacetate** **(to be given IV in 10% glucose)**	**L-arginine hydrochloride**^**a**^ **(to be given IV in 10% glucose)**	**N-carbamylglutamate** **(only oral/enteral drug)**
Undiagnosed patient ^b^	250mg/kg as a bolus in 90–120 min, then: maintenance 250-500mg/kg/d ^c^ (if >20 kg body weight, 5.5 g/m2/d)	250mg/kg as a bolus in 90–120 min, then maintenance: 250-500mg/kg/d ^c^ (1.2mmol/kg/d)	250(−400) mg/kg (1-2mmol/kg) as a bolus in 90–120 min, then maintenance 250 mg/kg/d (1.2mmol/kg/d)	100mg/kg bolus per NG tube then 25–62.5mg/kg every 6h
NAGSD	same	–	250 mg/kg (1.2mmol/kg) as a bolus in 90–120 min, then maintenance 250mg/kg/d (1.2mmol/kg/d)	same
CPS1D & OTCD	same	250mg/kg as bolus in 90–120 min, then maintenance: 250(−500) mg/kg/d ^c^	same	–
ASSD	same	same	same	–
ASLD ^d^	same	250mg/kg as bolus in 90–120 min, then maintenance: 250mg/kg/d ^c^	200-400mg/kg (1-2mmol/kg) as bolus in 90–120 min, then maintenance 200-400mg/kg/d (1-2mmol/kg/d)	–
ARG1D ^e^	same	–	AVOID	–
HHH syndrome	same	250mg/kg as bolus in 90–120 min, then maintenance: 250mg/kg/d ^c^	–	–

In severe acute decompensation **both** sodium benzoate and sodium PBA/phenylacetate should be given in parallel as “*ultima ratio”*. In less severe cases, a stepwise approach with initial sodium benzoate and if hyperammonemia persists or worsens, the addition of sodium PBA/phenylacetate can be chosen. The doses given can be used at the start of treatment but must be adapted depending on plasma ammonia and amino acids levels. Maximal daily dosages of sodium benzoate, sodium PBA and L-arginine should not exceed 12g for each of the three drugs. *Grade of recommendation, D*.

^a^ If citrulline is given, there is usually no need for concomitant use of L-arginine.

^b^ In undiagnosed patients, consider additional use of carnitine 100mg/kg IV, hydroxycobalamin 1mg IM/IV, and biotin 10mg IV/PO.

^c^ If on hemodialysis/hemodiafiltration maintenance doses should be increased to 350mg/kg/day (or proportional increase for body surface-based dose calculation).

^d^ In ASLD, L-arginine therapy for acute decompensations might be sufficient for some patients.

^e^ The risk for acute hyperammonemic decompensation is low in ARG1D.

#### Statement #16. Grade of recommendation: C

Administration of ammonia scavengers, of *L-*arginine or *L-*citrulline and in NAGS deficiency, of carbamylglutamate, is highly valuable for treating acute hyperammonemic decompensation. The potential toxicity of repeated boluses or high doses of benzoate or phenylacetate should be taken into account.

### Management of a neonate at risk of a UCD at birth. Grade of recommendation: D

The present recommendations are adopted from the *‘BIMDG Management Protocol of a baby at risk of a urea cycle disorder’* ( http://www.bimdg.org.uk/). The metabolic history of the previous index case including specific diagnosis and pedigree analysis (same father?) should be taken. Measures for minimising delivery stress should be considered, planning delivery in a hospital and having rapid access to a specialised metabolic unit. If there is risk of early neonatal presentation, it is recommended to transfer the newborn to the neonatal unit immediately after birth. Within 30 min 10% glucose infusion (4 ml/kg/h) should be started and after 4 symptomless hours, protein-free feeds should be given, along with reduction of glucose infusion, and administration of PO 6-hourly 50 mg/kg of both sodium benzoate and L-arginine. Plasma ammonia should be measured at 6 h and if <80 μmol/L re-assay every 6 h is recommended, while protein-free feeds are continued 3-hourly, changing to normal feeding after 24 h if ammonia remains <80 μmol/L. In contrast, if ammonia reaches 80–150 μmol/L, a preanalytical problem should be excluded, and re-assay in 4 h is recommended. If it remains in this range, monitoring should continue at 6-hourly intervals, whilst stopping protein containing feeds and continuing glucose infusion. With ammonia >150 μmol/L or if the baby becomes unwell, plasma ammonia levels should be repeated immediately. Feeds **must be stopped** (but protein-free nutrition should not exceed 24–48 hours) and actions taken as per Tables 4 and 5. Plasma amino acids should be measured (quantitatively) urgently at 12 h of age regardless of the plasma ammonia concentration. At the same time, blood samples for molecular genetic diagnosis should be collected and sent (not cord blood because of potential maternal contamination).

If a previous sibling had a late onset presentation, the glucose infusion should only be started if the birth was complicated. Otherwise, infant formula providing ≤2g protein/kg/d or demand breast feeding should be started, and plasma ammonia and amino acids (quantitatively) measured at 24 h of age. If ammonia is <150 μmol/L, re-analysis is indicated after 12 h but if ammonia >150 μmol/L or if the baby becomes unwell ammonia should be repeated immediately and actions taken as per Tables 4 and 5. If at 48 h ammonia is <80 μmol/L, milk feeds should continue (providing ≤2g protein/kg/d), whereas if ammonia is 80–150 μmol/L between 24 to 48 h and the baby is well, re-analysis at 12-hourly intervals is indicated. The results of quantitative plasma amino acids must be obtained, and feeds changed to a protein-free formula. OTCD female patients have a low risk of symptomatic hyperammonemia in the newborn period.

### Extracorporeal detoxification

Continuous veno-venous hemodiafiltration (CVVHDF) should be started in neonates and children who have ammonia levels of >500 μmol/L but at lower levels if there has been an inadequate response to medical management after 4 hours (this being the estimated time for preparing dialysis, including vascular access [103]). The modality of dialysis is partially determined by the local facilities, although hemodialysis in neonates is difficult and should only be performed by experienced teams and peritoneal dialysis is much less effective than other methods [56].

#### Statement #17. Grade of recommendation: C-D

In neonates and children with symptomatic hyperammonemia, dialysis should be carried out when ammonia exceeds 500 μmol/L or when there is no response within four hours after starting medical treatment.

 In adults with acute decompensations, hemodialysis (HD) or continuous veno-venous hemofiltration (CVVH) are first-line treatments because of wider availability and low risk, even if the diagnosis is not yet certain. Given the susceptibility of adults to develop intracranial hypertension and cerebral edema with hyperammonemia, dialysis should be started quickly, even before transfer to a specialised center, if ammonia exceeds 200 μmol/L. Although the decision to dialyze should consider also the existence of co-morbidities and the availability of and the tolerance to medications.

#### Statement #18. Grade of recommendation: C-D

Extracorporeal detoxification is the first line treatment in acute hyperammonemic decompensations in adults.

HD is intermittent and gives the highest ammonia extraction, but some patients experience acute relapses after its discontinuation. Due to frequent technical and hemodynamic complications related to HD in infants [104] ammonia removal in neonates may be more effective with CVVH. CVVHDF is a continuous procedure with excellent ammonia clearance and usually well tolerated in small infants, for whom it is the preferred method of dialysis [56,99,105].

#### Statement #19. Grade of recommendation: C

The method of choice for ammonia detoxification is hemodiafiltration. Peritoneal dialysis is a far less effective method. Exchange transfusion should not be used.

### Dietary management of acute decompensation

The aim is to minimise protein (nitrogen) intake temporarily and prevent endogenous protein catabolism whilst providing enough energy to meet metabolic demands [106]. If the patient starts to feel unwell but oral feeding is still possible, a high energy, protein free feeding regimen which is based on glucose polymer (Table 6) should be immediately given. In cases of impaired consciousness or vomiting, glucose should be infused (plus IV insulin at a starting dose of 0.05 units/kg/h in case of hyperglycemia) as soon as possible to maximise energy intake [107]. IV lipids can also be given to increase energy, at a dose of (1)-2-(3) g/kg/d. Following improvement of hyperammonemia reintroduction of protein/essential amino acids (EAAs) should not be delayed beyond 24 to 48 h. If the patient cannot be fed enterally, IV amino acids should be commenced, increasing daily to the required amount.

**Table 6:** Emergency regimen for protein-free feeding in infants and children

**Age**	**Glucose polymer concentration (% carbohydrates)**	**Energy/100ml**		**Suggested daily intake**	**Feeding frequency**
		**kcal**	**kJ**		
up to 6 m	10	40	167	150ml/kg	2 to 3 hourly oral/bolus
7-12 m	10-15	40-60	167-250	120ml/kg	day and night
1 y	15	60	250	1200ml	or continuous tube
2-9 y	20	80	334	Estimate as indicated^a^	feeds using enteral
>10 y	25	100	418	Estimate as indicated^a^	feeding pump

Adapted from Clinical Pediatric Dietetics [106]. *Grade of recommendation, C-D*.

^a^ For children >10 kg emergency regimen fluid requirements can be calculated as:

11–20 kg: 100ml/kg for the first 10 kg, plus 50ml/kg for the next 10 kg.

>20 kg: 100ml/kg for the first 10 kg, plus 50ml/kg for the next 10 kg, plus 25ml/kg thereafter up to a maximum of 2500ml/day.

* Enteral feeding * should be re-started as soon as possible. It may initially be protein-free (Table 6). Transient nasogastric (NG) feeding may be necessary to achieve an adequate intake. Enteral fluids should be increased as IV fluids are decreased. The sodium given with nitrogen scavengers (see Table 4) should be considered in calculating the total electrolyte intake. Osmotic diarrhoea because of over-concentrated feeds should be avoided. In practice, once the blood ammonia has fallen to <100 μmol/L protein is usually re-introduced, to the ‘safe level of intake’ (see below section on low protein diet) or the child’s usual intake over 2–4 days, whilst monitoring ammonia. If ammonia levels increase during protein re-introduction, special mixtures of EAAs for UCDs can be used instead of or in combination with natural protein. The energy intake should aim to provide about 120% of age-adjusted requirements.

#### Statement #20. Grade of recommendation: D

For treatment of acute hyperammonemia it is crucial to promote and maintain anabolism by infusing high-dose glucose plus lipids (if a fatty acid oxidation disorder has been excluded). Protein should be reintroduced when ammonia returns to <100 μmol/L. Ideally the period of protein-free nutrition should not exceed 24–48 hours.

## Long-term management of UCDs

The goals of long-term management are to achieve normal development and to prevent hyperammonemia, whilst providing a good quality of life and avoiding side-effects and complications [12,16,108]. It is based on:

· low protein diet

· essential amino acids supplementation

· vitamin and mineral supplementation

· medications to increase waste nitrogen excretion

· caring for special situations and provision of emergency regimen in intercurrent illnesses

· liver transplantation for selected patients.

A detailed, written day to day treatment plan and emergency regimen (see dietary management of acute decompensation above), including instructions on when and how to contact the metabolic team or the local hospital should be given to parents/caregivers and to the child’s nursery or school.

### Low-protein diet

This mainstay of long-term management is based upon minimising the nitrogen load on the urea cycle. The amount of natural protein tolerated by each patient must be individually determined and by titration against ammonia. The FAO/WHO/UNU 2007 have set *‘safe levels of protein intake’*[109] calculated as an age-adjusted mean + 2-standard deviations (SD) (Table 7) and can be used as a guide. Lower protein intakes may still be adequate [106] but individualised over-restriction may compromise growth and cause metabolic instability [47,110]. If the intake is too low, EAA supplementation may be indicated (see below). An adequate energy supply must also be guaranteed to prevent catabolism and consequent hyperammonemia. The FAO/WHO/UNU 2007 Report [109] (summarised in Table 7) can be used as a guide to energy intakes. Patients with reduced mobility will have lower energy expenditures and therefore lower energy requirements. To ensure sufficient energy and protein intake NG tube or gastrostomy feeding (see below) may be necessary. Ideally the diet should be provided by normal food, combining low and high biological value protein foods, naturally low or protein free foods, additionally for some patients, specially manufactured low protein foods. The daily protein intake should if possible be divided equally between three meals and some for snacks. Long fasts should be avoided, and a pre-bed snack given to reduce the risk of overnight catabolism. Regular monitoring of protein intake, growth and clinical status are essential, because protein requirements and tolerance vary with age, growth velocity, disorder nature and severity and frequency of intercurrent illnesses. Compared with older children, metabolic control may be easier in early infancy, when their rapid growth results in increased protein tolerance [16,109].

**Table 7:** **Selected values from FAO/WHO/UNU safe levels of protein intake and energy requirements of children and adults, as well as during pregnancy and lactation, for the healthy population**[109]

**PROTEIN INTAKE**			**ENERGY REQUIREMENTS**				
**Age**	**Intake**		**Age**	**Females**	**Males**	**Females**	**Males**
***months***	***g/kg bw/day***		***years***	***kJ/kg bw/day***		***kcal/kg bw/day***	
1			0.5	340	335	81.3	80.0
2	1.50		2.5	334	348	79.8	83.2
3	1.36		5.0	305	315	72.9	75.3
6			10	248	275	59.3	65.7
12	1.14		15	193	230	46.1	55.0
** *years* **							
1.5	1.03		**Adults, moderate activity level, 70kg body weight**				
2	0.97						
3	0.90		18-29	159	183	38.0	43.7
4-6	0.87		30-59	148	175	35.4	41.8
7-10	0.92						
	**Females**	**Males**	**Adults, moderate activity level, 50kg body weight**				
*years*							
11	0.90	0.91	18-29	180	212	43.0	50.7
12	0.89	0.90	30-59	183	212	43.7	50.7
13	0.88	0.90					
14	0.87	0.89	**Pregnancy total extra energy requirements**				
15	0.85	0.88	** *trimester* **	** *kJ/day* **		** *kcal/day* **	
16	0.84	0.87	1st	375		90	
17	0.83	0.86	2nd	1200		287	
18	0.82	0.85	3rd	1950		466	
> 18	0.83	0.83					
			**Lactation total extra energy requirements**				
**Pregnancy:**			** *months* **	** *kJ/day* **		** *kcal/day* **	
**Total extra protein intake**							
** *trimester* **	** *g/day* **		1-6	2800		669	
1st	1		>6	1925		460	
2nd	10						
3rd	31						
**Lactation:**							
**Total extra protein intake**							
** *months* **	** *g/day* **						
1- 6	19						
> 6	13						

bw: body weight.

#### Statement #21. Grade of recommendation: C-D

Dietary treatment is a cornerstone of therapy. This warrants the particular expertise of a specialist metabolic dietitian to finely balance nutritional requirements with metabolic stability. The FAO/WHO/UNU recommendations can be used to guide the protein and energy requirements.

### Supplementation of EAAs and other essential nutrients

EAA supplementation is required when natural protein tolerance is too low to achieve normal growth and metabolic stability. A reasonable approach [111] is to provide 20-30% of the total protein intake as EAA supplements, except in ARGD1, when supplements of EAA can, if necessary, be increased to 50% of total protein because of the severity of protein restriction (see below). EAA’s should be given as an equally divided dose with 3 or 4 main meals to enhance utilisation and prevent nitrogen overload. Plasma levels of branched-chain amino acids (BCAAs, a subgroup of the EAAs) are decreased in patients taking high doses of sodium PBA [112], suggesting the potential need of BCAA supplementation [113,114]. It has been advocated that EAA supplements should be rich in BCAAs but not in the neurotransmitter precursor amino acids tryptophan, phenylalanine and tyrosine [114]. BCAA supplements are available as single amino acids or complete mixture and as per EAAs given as a divided dose.

#### Statement #22. Grade of recommendation: C

Essential amino acids and branched-chain amino acid supplements can form part of the total protein intake if natural protein tolerance is very low and the patient is not metabolically stable.

UCD patients on low protein diets are at risk of vitamin and mineral deficiencies particularly of Fe, Zn, Cu, Ca and cobalamin [106] and supplementation will be required. Additionally, they may be at risk of essential fatty acid (EFAs)/long-chain polyunsaturated fatty acid deficiencies [115,116]. These can be provided in the diet from EFAs/ (LCPUFAs)-enriched infant formulas, oils rich in polyunsaturated fatty acids (e.g., walnut, rapeseed or sunflower oils) or as a separate supplement of docosahexaenoic acid and arachidonic acid.

### Practical aspects of dietary management of low-protein diet

The main protein source for infants should be either breast feeding or standard infant formula.

Exclusive demand * breast feeding *[117,118] is possible but this needs close analytical/clinical monitoring, and if necessary protein intake can be limited by giving protein-free infant formula prior to breast feeds. In * bottle feeding, * the protein is given as limited volume of infant formula (sometimes together with EAAs) and divided evenly between the daily feeds. Each protein feed is followed by a nutritionally complete but protein-free formula to appetite. Combined the feeds should provide the normal requirement for all nutrients. Normal * weaning * practices can be followed by first introducing fruits and very low protein vegetables [106] then gradually replacing gram by gram of protein from breast milk/infant formula with protein-containing foods. Parents need careful training and close supervision in calculating the protein content of food, to allow this changeover from feeds to food. Vitamin and mineral supplementation should be started as weaning progresses and less milk feeds are taken. Feeding problems do occur in patients with UCDs and can cause inadequate nutrient intake and metabolic instability. For some it is possible to give concentrated energy supplements such as glucose polymer and/or fat emulsions but tube feeding may be essential (see below). The diet must be adjusted regularly throughout * childhood *. Close monitoring is generally needed in * adolescence * because of the tendency to poor compliance, increased appetite and nutrient requirements of puberty thus an increased risk of metabolic instability. Late onset patients who are on a self-selected low-protein diet generally require vitamin and mineral supplements since they are likely to be deficient in cobalamin, iron and calcium, at least. Adolescent and * adult patients * should know that unless treated by liver transplantation they will need life-long, low-protein diet and regular dietary assessments. * Pregnancy and lactation * in UCD patients [119,120] requires addressing special nutritional needs (Table 7), avoiding protein deficiency and close monitoring of metabolic status, particularly during and for five days post delivery to ensure prompt recognition and treatment of any hyperammonemia. * In all patients *, the last meal of the day should aim to provide ~25% of the daily intakes of energy, natural protein, (EAAs if taken), citrulline and/or arginine (see below) to minimise catabolism during the overnight fast and to optimise urea cycle function.

#### Statement #23. Grade of recommendation: C-D

The changing needs of the different stages of normal development and of special situations such as pregnancy and lactation necessitate careful, individualised dietary management plans. Parents and patients should be trained on food protein calculation and provision of adequate energy and nutrient intake. They need to be aware of the need for life-long dietary treatment and regular dietary assessments.

* Tube feeding * is essential in cases of: i) inability to suck or swallow because of neurological handicap or developmental delay; ii) severe vomiting, reflux or retching; iii) poor appetite and/or refusal of food, EAA supplement or drugs; iv) emergency management during intercurrent illnesses. In acute episodes, nasogastric tubes may help expedite the transfer from parenteral to enteral nutrition. If tube feeding is needed in the long-term, gastrostomy is recommended despite the lack of controlled studies and the risk of tube feeding-dependence [107]; oral food and fluids can be offered in addition unless swallowing is compromised. The pattern of tube feeding (bolus, continuous, during the day and night) should be based on feed tolerance and consideration to the patient’s daily routine.

#### Statement #24. Grade of recommendation: C-D

Tube feeding may be needed to: achieve nutritional adequacy, administer medications and supplements, prevent catabolism and maintain metabolic stability.

### Pharmacotherapy for long-term treatment

The drugs used for acute detoxification are also utilised as oral preparations for long-term treatment. Given the multiplicity of oral preparations, their prescription should be unambiguous [3,75].

#### Statement #25. Grade of recommendation: D

Written drug treatment sheets should be provided to parents, pharmacists and persons involved in patient care.

Among the * nitrogen scavengers, sodium benzoate *, although not a registered drug, has been used for decades in UCDs, whereas * sodium PBA * is a licensed drug that has more recently been introduced. Although in theory phenylbutyrate can scavenge twice as much nitrogen as benzoate [83,121], the superiority in vivo of PBA has been questioned [122]. Many European centres as well as a majority of guideline group members prefer sodium benzoate because PBA is a histone 1,2 deacetylase inhibitor [123] that could have as yet unrecognized long-term side effects. Nevertheless, at the recommended doses (Table 8; [3,73]) both nitrogen scavengers appear safe. Sodium benzoate is toxic only at plasma levels >2 mmol/L [124]. Sodium PBA causes menstrual dysfunction/amenorrhea in ~25% of postpubertal females [101], and can decrease appetite, disturb taste and cause disagreeable body odour. Also it may deplete BCAAs and increase the risk of endogenous protein catabolism [112]. Since both scavengers are esterified with CoA, they could cause acetyl-CoA depletion, resulting in mitochondrial dysfunction [125] and decreased NAG synthesis. Low albumin levels have been reported in some sodium PBA-treated patients [112,113,126], possibly because of decreased leucine and glutamine availability.

**Table 8:** Dosages of peroral drugs for long-term treatment of UCDs

**Disorder**	**Sodium benzoate**^**a**^	**Sodium PBA**^**a,b**^	**L-Arginine**^**a**^** (hydrochloride or free base)**	**L-Citrulline**^**a**^	**Carbamyl- glutamate**^**a**^
NAGSD	–	–	–	–	10–100 mg/kg/d
CPS1D	≤ 250mg/kg/d^c,d ^maximum: 12g/d	<20 kg: ≤250mg/kg/d^c,d ^>20 kg: 5g/m2/d maximum: 12g/day	<20 kg: 100-200mg/kg/d^c^(0.5-1mmol/kg/d) >20 kg: 2.5-6g/m2/d maximum: 6g/d	100-200mg/kg/d^e ^maximum: 6g/d	–
OTCD	same	same	same	same	–
ASSD	same	same	<20 kg: 100-300mg/kg/d^c,d^>20 kg: 2.5-6g/m2/d maximum: 6g/d	–	–
ASLD	same	–	same	–	–
ARG1D	same	same	–	–	–
HHH syndrome	same	same	<20 kg: 100-200mg/kg/d^c ^>20 kg: 2.5-6g/m2/d maximum: 6g/d	same	–

All medications should be divided into three to four equal daily doses taken with meals and distributed as far as possible throughout the day. *Grade of recommendation, C-D.*

^a^100 mg of each drug correspond to the following amounts in mmols: 0.694 sodium benzoate; 0.537 sodium PBA; 0.475 arginine hydrochloride; 0.574 arginine base; 0.571 citrulline; 0.532 carbamylglutamate.

^b^ Sodium PBA was considered of second choice for long-term treatment by most guideline group members. It should be given together with sodium benzoate in patients in which benzoate alone is not enough.

^c^ Serum/plasma levels of benzoate/PBA and plasma levels of arginine (aim are fasting levels of 70–120 μmol/L) should be monitored to adjust dosages and in case of high or repeated doses.

^d^ In some patients higher doses are needed, according to expert advice. For PBA, FDA and EMA approved doses are 450-600mg/kg/d in children of <20kg, and, above 20 kg bw, 9.9-13 g/m^2^/d.

^e^ Citrulline may be preferable. When given no need for concomitant use of L-arginine.

#### Statement #26. Grade of recommendation: C

To avoid mucositis or gastritis, oral sodium benzoate and sodium PBA (as granules, tablets, or undiluted liquid preparations) should be taken 4-times daily during meals with abundant fluids*.*

Hypokalemia can develop after repeated boluses of scavengers and in long-term treatment, resulting from increased renal loss of K^+^. Metabolic acidosis has been observed with high doses of both scavengers [100,101]. The doses given in Table 8 for these scavengers reflect a literature- [1,100,101] and experience-based consensus. Despite anecdotal evidence of successful pregnancies of women taking sodium PBA [101], sodium benzoate appears potentially a safer choice during pregnancy.

#### Statement #27. Grade of recommendation: C

Use of nitrogen scavengers appears safe at the recommended doses.

#### Statement #28. Grade of recommendation: D

Information on nitrogen scavenger therapy during pregnancy is scarce. There is a compelling need for a registry to collect all unreported cases.

* L-arginine * is an essential amino acid in all UCDs because of its impaired synthesis (except in ARG1D) and must be supplemented as such or as its precursor * L-citrulline *[3,127-130]. In ASSD and ASLD, citrulline and particularly ASA, respectively, serve as vehicles for nitrogen removal via their excretion in the urine and thus arginine administration reduces the frequency of hyperammonemic episodes [3,128-130]. In these patients fasting plasma arginine concentrations should be about 70–120 μmol/L [3]. In NAGSD, CPS1D, OTCD and the HHH syndrome L-citrulline may be supplemented instead of L-arginine but there are no studies comparing the efficacy of the two.

#### Statement #29. Grade of recommendation: C

All UCD patients should be monitored for plasma arginine levels. Except in ARG1D, most will need L-arginine or L-citrulline supplementation.

* N-carbamyl-L-glutamate, * also called carbamylglutamate or carglumic acid is a deacylase-resistant NAG analogue that is taken up enterally and replaces NAG in the activation of CPS1, thus being the definitive therapy for NAGSD [131-133]. It is licensed in Europe by the European Medicines Agency (EMA) and approved in the US by the Food and Drug Administration (FDA). It can also be used as an emergency medication and a tool for diagnosis of NAGS deficiency in neonatal hyperammonemia [134]. Despite the lack of controlled studies, its use should be considered in severe hyperammonemic decompensations. In addition to UCDs, N-carbamyl-L-glutamate is licensed by the EMA for treatment of hyperammonemia in some organic acidurias [135]. There are no reports on long-term safety or on adverse effects of this drug other than high dose-triggered Chinese restaurant syndrome [133].

#### Statement #30. Grade of recommendation: C-D

N-carbamyl-L-glutamate is the first line medication for treatment of NAGSD and may also be used as an emergency drug during acute neonatal hyperammonemia of unknown etiology.

UCD patients may have carnitine deficiency [136-138] because low protein diets can be low in carnitine and nitrogen scavengers also conjugate with carnitine. Plasma carnitine should be monitored in UCD patients and severe carnitine deficiency treated with carnitine supplements.

#### Statement #31. Grade of recommendation: D

In cases of proven plasma carnitine deficiency 25–50 mg/kg/d carnitine should be given.

 Although neomycin and metronidazole have been used to decrease the load of ammonia-producing bacteria in the colon, there is currently no good evidence to support its use.

### Caring for special situations and emergency regimen in intercurrent illnesses

If there is an immediate risk of hyperammonemia as a consequence of an intercurrent illness or other event, the * emergency regimen * (Table 6) should be started without delay, even at home. If there is no prompt improvement the metabolic unit should be contacted urgently (see above section on acute management).

Despite the potential risks of fever, * vaccinations * have not been identified as causes of metabolic decompensation [139,140].

#### Statement #32. Grade of recommendation: B

Vaccinations do not substantially increase the risk of decompensations and are recommended at the same schedule as for healthy children. If temperature exceeds 38°C antipyretic treatment should be started.

To minimise decompensation risks [108], elective surgery should be undertaken when the patient is completely well including no minor intercurrent illness and has normal ammonia/amino acid levels. Surgery should only be carried out in centres prepared for dealing with acute hyperammonemic decompensations. The day before surgery drug treatment should be switched to IV and glucose (10%) infused to ensure anabolism. The patient should be first on the day’s surgical schedule. Midazolam, s-ketamine, fentanyl and isoflurane in combination with surgical field infiltration with ropivacaine have been reported [141] as safe anesthetic agents. Post-surgery, close monitoring of the clinical status and ammonia levels is required, shifting to oral medications and diet and stopping the IV glucose administration only if the patient is well and metabolically stable.

#### Statement #33. Grade of recommendation: D

Elective surgery in UCD patients should be performed in centres with a metabolic department including emergency treatment options for hyperammonemia.

## Liver transplantation for UCD patients

### The role of liver transplantation

Liver transplantation, reported with all UCDs except NAGSD and the HHH syndrome, is curative as far as enzyme deficiencies are concerned and allows termination of the low-protein diet and regular alternative pathway therapy, but does not reverse established neurological sequelae, [142]. Transplanted patients require immunological therapy and long-term follow-up. The inability to synthesise arginine extrahepatically persists, but this metabolic aberration has no recognized clinical impact. Reported post-transplant survival in OTCD and in non-UCD patients has been the same [143,144], attaining ~95% at one year and ~90% at 5 years in large pediatric liver transplantation programs [145] with “good” or “excellent” self-reported quality of life at 6–121 months post transplant [144,146-149]. Thus, liver transplantation offers severely affected UCD patients a better alternative in terms of quality of life than medical treatment. Since the pre-existing neurological damage appears not to reverse [150-157], it is essential to prevent endogenous catabolism and hyperammonemia prior to and during liver transplantation. Transplantation during acute encephalopathy and/or acute liver failure has been carried out [34], but these high risk situations require case-by-case discussion of the need to transplant.

#### Statement #34. Grade of recommendation: C

The only available curative treatment for UCDs is liver transplantation, allowing return to a normal diet and stopping drug administration. Ideally it should be performed in a patient without severe neurological damage whilst in a stable metabolic condition. In patients with important neurological acute disease or sequelae, liver transplantation has to be discussed.

### Indications and age for liver transplantation

Liver transplantation should be deferred, if possible, until 3 months of age and/or 5 kg body weight to avoid the increased complications and lower survival rates observed when performed before attaining this age and weight [158,159]. Ideally, it should be done before 1 year of age for best neurological outcome [155], especially in CPS1D, in OTCD males, in patients with recurrent metabolic decompensations despite treatment or when treatment compliance is poor. In selected cases presenting with poor treatment compliance, diet-induced growth retardation, poor school attendance, altered psychological status or problems with familial and social integration, transplantation may be considered later, sometimes even during adolescence. OTCD females presenting symptoms in the first 2 years of life have a second lethality peak at 12–15 years of age [160] and thus they should also be considered for liver transplantation.

#### Statement #35. Grade of recommendation: D

Liver transplantation should ideally be carried out before irreversible neurological damage and/or repeated crises, generally between 3 and 12 months of age. It may be considered in all patients (excepting NAGSD) with severe neonatal onset. It is also indicated for patients with progressive liver disease (e.g. ASLD), for those suffering recurrent metabolic decompensations and hospitalisations despite medical therapy, as well as those with poor quality of life.

### Transplant type, donor and ethical issues

Standard orthotopic liver transplantation (OLT) is preferred to auxiliary liver transplantation because it has fewer complications [144]. Transplantation of liver lobes from living relatives can reduce waiting times and gives results comparable to those obtained with cadaveric organs [145], albeit that it entails a small risk for donors [161]. Heterozygosity for the disease in the living related donor is not a problem, except in OTCD females, although asymptomatic OTC heterozygotes have been successful donors [162,163]. Decisions on whether or not to perform liver transplantation entail ethical considerations requiring individualised decision, in particular when the child is already handicapped or when living donor liver transplantation is considered.

#### Statement #36. Grade of recommendation: D

The recommended procedure is orthotopic liver transplantation. Ethical issues concerning the recipient and the risk for living donors render essential careful pre and post transplantation counselling.

## Patient monitoring

Medically treated UCD patients require lifelong monitoring including anthropometric data, biochemical tests, dietary and drug review, history of intercurrent illnesses and use of the emergency regimen. Visit intervals should be individualised on the basis of age, growth, severity, metabolic stability and compliance with diet and drug therapy. Young and severely affected patients may need monitoring every 3 months, whilst annual reviews may be enough for older or less affected patients.

* Clinical monitoring * should address: growth and head circumference, inspection for thin sparse hair, hair loss, skin rashes and other signs of protein/vitamin deficiency, neurological examination and neurocognitive development, liver size and structure (by ultrasound scan). Regular * dietary assessments * are also essential, either by recall, or when nutritional or compliance problems are suspected, by recording quantitatively diet, supplements and drugs taken in the 3–5 days preceding the visit. The diet needs to be adjusted for age and growth (see below) [17,47,106]. Since low protein diets may increase the risk of osteoporosis, bone density monitoring from time to time may be advisable [164,165].

### *Statement #37. Grade of recommendation: D*

Clinical, biochemical and nutritional monitoring are essential and should follow an individualised plan.

* Laboratory monitoring * must include * plasma ammonia * determination in venous samples (target level <80 μmol/L [16,100]). Spurious elevations can be due to poor sample processing, tourniquet, crying, struggling or convulsions [166]. Sequential determination of ammonia for 24 h, including preprandial, postprandial and fasting samples, is carried out in some centers but its value is uncertain. A reflectometric device (Ammonia Checker™ II) [167] exists that allows bedside approximate assessment of ammonia in 3 min using 20 μl of venous blood (but upper limit of detection of this device is 280 μmol/L). It is not recommended for home use since capillary samples are unsuitable because cell contents and sweat contamination can lead to false high values.

The * plasma amino acid profile * should be determined to ensure that enough arginine/citrulline and EAAs/BCAAs are supplied. Arginine should be in the high normal range and EAAs and BCAAs in the normal ranges. Rising glutamine may indicate impending hyperammonemia. Arbitrarily, glutamine levels are considered tolerable when they do not exceed 1000 μmol/L [16,73,100,168]. Since glutamine levels change with the fasting/feeding status, being highest after the overnight fast [169], sampling should be standardised, ideally 3–4 h after the last meal [170,171]. It is important to consider growth rate, age, actual protein intake and frequency of use of the emergency regimen when interpreting plasma amino acids as low EAA levels do not always necessitate an increase in protein. Decreasing the nitrogen scavenger dose may also help to increase BCAAs concentrations.

* Other blood assays * can include determination of vitamins (including cobalamin), minerals, trace elements, carnitine, ferritin, cholesterol and triglycerides in plasma and of EFAs in red blood cells and plasma. Blood urea levels are of little value since they depend mainly on arginine intake and tubular urine flow rate. Blood sodium benzoate and/or sodium PBA/phenylacetate assays may be helpful to prevent toxicity in patients receiving high dosages or repeated boluses [172].

* Urine determinations *: ketone bodies (done at home after instruction of parents) might indicate a catabolic situation. Hippurate quantitation allows assessing compliance with benzoate treatment. Amino acid profiling is not recommended; the value of measuring orotate and orotidine excretion is dubious, although increasing orotate excretion should reflect greater ammonia load and carbamoylphosphate accumulation.

### *Statement #38. Grade of recommendation: D*

Venous ammonia and amino acid levels should be monitored, aiming at levels of ammonia <80 μmol/L, glutamine <1000 μmol/L, arginine in the high normal range and EAAs and BCAAs in the normal range. Rapid reflectometric ammonia assay in venous blood is useful, provided it’s limitations are known by the user.

* Neuroimaging * should assess possible neuroanatomical alterations, providing *"information about the timing, extent, reversibility and possible mechanism of neural injury .... and can be used as an adjunctive measure to predict clinical and neurocognitive outcome”*[173]. Magnetic resonance imaging (MRI) should ideally be performed systematically between days 1 and 4 of each coma or stroke-like episode, to follow the changes in the apparent diffusion coefficient (ADC). If general anesthesia is not needed, it should be repeated at approximately 2 year-intervals to allow correlation of motor/language/cognitive development with anatomic changes. MRI sequences should include diffusion tensor imaging, axial T2 and FLAIR, sagittal and axial T1, and magnetic resonance spectroscopy (MRS) [173-175]*.* In acute presentations, diffuse cerebral edema is seen as areas of cortex and the underlying white matter presenting abnormal signal intensity, infarct-like aspect and restricted diffusion. These areas are often multiple in one or both hemispheres and are asymmetrical [176,177]. Basal ganglia involvement is revealed on T2-weighted images by high intensity signals in the caudate nucleus, putamen, and/or globus pallidus. The deep sulci of the insular and perirolandic regions may also display T1 shortening [176]. MRS can reveal highly elevated brain glutamine levels [178,179]. These elevations are helpful to detect subtle changes in OTC females [174,175]. The thalamus, brainstem, the occipital region and the cerebellum tend to be relatively spared and a few months after acute hyperammonemia a very moderate residual hypersignal on the insula and rolandic region may be observed [13,176]. Chronic hyperammonemia may convey defective myelination and progressive cerebral atrophy, with nonspecific punctate white matter hyperintensities seen in some patients [173,175].

### *Statement #39. Grade of recommendation: D.*

To help predict clinical and neurocognitive outcome it appears desirable to perform magnetic resonance imaging early on in each coma or stroke-like episode, and at 2-year intervals. Investigations should include diffusion tensor imaging, axial T2 and FLAIR, sagittal and axial T1 and magnetic resonance spectroscopy.

### Cognitive outcomes and psychosocial issues in UCDs

Cognitive outcome appears to be poor for neonatal onset patients, and less so for late-onset patients. Thus, a study with 26 survivors of neonatal hyperammonemic coma found that ~79% were mentally retarded and that the mean IQ value for the entire cohort was only 43 [180], whereas another study found that ~50% of 33 neonatal patients but only ~25% of 59 late-onset patients presented moderate to severe intellectual disability [91]. The level and duration of hyperammonemia appear key determinants of the outcome [13]. It was reported [4] that no UCD patient having had >300 μmol/L initial or >480 μmol/L peak plasma ammonia exhibited normal psychomotor development. Nevertheless, another study [85] found average or above average IQ values in 33%, 40% or 66% of the ASSD, ASLD and OTCD patients studied, respectively, although the predominance of late-onset OTCD presentations (81%) in that study might account for the good outcome of these patients [91]. The importance of assessing not only IQ and development but also the specific patterns of neuropsychological strengths and weaknesses was illustrated by the finding in late-onset OTCD patients having normal IQ of deficits of motor planning and execution [181] and a specific neurocognitive pattern that included weakness in fine motor dexterity/speed and a trend towards weakness in non-verbal intelligence, visual memory, attention/executive skills and mathematics, although verbal intelligence, memory, learning and reading were strong [91]. In any case, clinically asymptomatic OTC heterozygotes outperformed symptomatic heterozygotes and the performance improved with higher levels of residual urea synthesis activity, assessed by stable isotope studies, whereas neither the allopurinol test results nor the mutation type correlated with neuropsychological performance in these patients [26].

#### Statement #40. Grade of recommendation: D

Neonatal onset and prolonged hyperammonemic coma predict severe impairment of future neurocognitive performance. Patients with milder disease or heterozygous carriers for OTCD may develop specific weaknesses in several executive functions even if the IQ is normal. Regular testing for IQ, development and specific abilities/weaknesses is recommended.

As is the general case for parents of chronically ill children [182], UCD patients/families can have low health-related quality of life (HrQuOL), with disease-imposed stresses perhaps amplified by delays in diagnosis and treatment resulting from poor general awareness of these diseases [183]. Thus, the recommendation for chronically ill patients and their families [184-186] of routine monitoring of emotional, behavioral and psychosocial parameters also applies to UCD patients, aiming at identifying and preventing psychosocial maladjustment [187]. Psychologists should be involved in patient care early after diagnosis to cope with initial anxiety and with later-developing psychological problems [186] and also to assess the cognitive level and neuropsychological functions of the patient.

#### Statement #41. Grade of recommendation: D

Health-related quality of life, anxiety, stress and psychosocial factors are important outcome parameters. Psychological management is an important component of the care of UCD patients and their families.

## Recommendations for specific disorders

Unless indicated otherwise, these disorders require the nutritional management given above. Table 8 gives specific recommendations for drug use in each disorder under situations of chronic management.

### NAGS and CPS1 deficiencies

**Figure 3:**
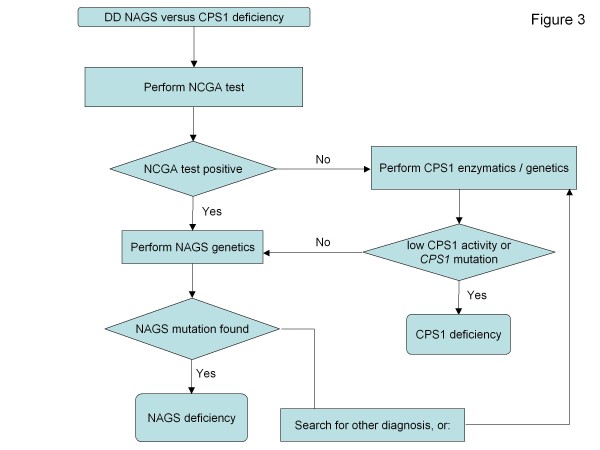
**Algorithm for the differential diagnosis of NAGSD and CPS1D.** NCGA: N-carbamoyl-L-glutamic acid [134]. *Grade of recommendation, C*.

These deficiencies have identical clinical and laboratory manifestations (Figures 2). Administered carbamylglutamate can replace the missing NAG in NAGSD, and thus a positive therapeutic response to carbamylglutamate indicates NAGSD [134]. However, one confirmed NAGSD patient was initially unresponsive to carbamylglutamate [188] whereas some CPS1D patients gave positive responses [189,190]. Confirmation of NAGSD or CPS1D requires mutation analysis or enzyme activity assays on liver biopsy when genetic diagnosis is inconclusive or rapid diagnosis is required. However, NAGS assay is highly specialised and is performed in few laboratories. Furthermore, abnormally low liver CPS1 activity has been reported in patients with genetically demonstrated NAGSD [29,191] and in the hyperinsulinism-hyperammonemia syndrome [192] (a defect involving glutamate dehydrogenase). Figure 3 provides an algorithm on how to proceed for differential diagnosis between NAGSD and/or CPS1D.

#### Statement #42. Grade of recommendation: D

Genetic analysis is virtually the only method of NAGSD diagnosis, since NAGS activity assay is not generally available.

#### Statement #43. Grade of recommendation: D

CPS1 assay can be faster than genetic analysis for confirming diagnosis of CPS1D, but it requires liver or intestinal mucosa. Genetic testing, although somewhat cumbersome because of the large size of the *CPS1* gene, allows non-invasive confirmatory diagnosis.

* NAGSD patients treated * with carbamylglutamate (available as oral medication) do not require protein restriction or other medications except during metabolic crises. The standard carbamylglutamate maintenance dosage of 100–200 mg/kg/day (given in 3–4 doses) [193], can be adjusted individually by progressive down-titration to the minimum dose required (as low as 10–15 mg/kg [194,195]) to maintain normal ammonia levels. Long-term outcome data are scarce [194,196] but the guideline group members know of twenty NAGSD patients that follow this treatment approach, all of whom are doing well. * CPS1D therapy * is as for other UCDs (Tables 5 and 8). L-citrulline administration would appear preferable over L-arginine since it should allow for the incorporation of one nitrogen atom into arginine and urea. However, L-citrulline is more expensive than arginine, it is not available for infusion and single reports indicate similar efficacy of L-citrulline and L-arginine [128].

#### Statement #44. Grade of recommendation: C

Mono-therapy with carbamylglutamate is the treatment of choice in NAGSD.

### OTC deficiency

This is the most prevalent of the UCDs [1-3,197]. Due to its X-linked nature, parental consanguinity is uncommon and males are more frequently affected [77,197]. While neonatal onset cases can generally be diagnosed reliably on biochemical grounds (elevated glutamine, very low/absent citrulline and elevated urinary orotic acid; see Figure 2), diagnosis may not be easy in the absence of a mutation in late onset cases. OTCD can present with acute liver disease/failure that generally recovers with metabolic management but that in some cases required urgent liver transplantation [34]. In the majority of female patients and in some male patients the mutation appears de novo and the mother is not a disease carrier [77]. Diagnosis is confirmed by identifying the mutation in DNA, an approach that permits detecting carrier females and affected fetuses [197] and that can help even genotype-based prognosis [77,198,199]. In ~20% of OTC patients the mutation is not identified with standard techniques [77]. Liver tissue-derived RNA studies [81] or oligonucleotide array comparative genomic hybridization (aCGH) [200] can be used to increase the mutation detection rate. Liver/intestinal mucosa OTC activity assay allows diagnostic confirmation in males, but gives less certain results in females because of the mosaicism caused by the lyonization process. Abnormal increases in urinary orotic acid/orotidine excretion after allopurinol administration (the allopurinol test) are also used to identify female carriers [201,202]. Although this test was reported initially as having 95% sensitivity and 100% specificity for OTCD [201,202], it is now clear that false-negatives and false-positives are common ( [203] and R. Santer and V. Valayannopoulos, personal communications) and that the test is not completely specific, since patients with ASSD, ARG1D, lysinuric protein intolerance or the HHH syndrome can give a positive result [204]. A modified protein loading test has been reported to have increased sensitivity [205] and to be less dangerous than older protein loading tests [12].

#### Statement #45. Grades of recommendation: C-D

When genetic analysis fails, OTC enzyme activity assay in liver or intestinal mucosa can help confirm the diagnosis. Pedigree analysis and allopurinol testing are also helpful, particularly for carrier identification if mutational analysis and enzyme assays are unavailable or negative.

The treatment and the considerations concerning preference for L-citrulline over L-arginine are as for CPS1D. Although many female patients have mild deficiency requiring little or no protein restriction, decompensation risks dictates for these females lifelong prevention and readiness for emergency measures, as well as twice a year monitoring, with determination of plasma ammonia and glutamine levels.

### ASS deficiency (citrullinemia type 1)

Diagnosis is generally straightforward (Figure 2) because of the combination of hyperammonemia and/or elevated plasma glutamine with very high levels of plasma citrulline and increased urinary orotic acid. ASSD can cause liver damage and even acute liver failure [38,39]. Confirmatory diagnosis by genetic or enzymatic methods enables prenatal testing [206] and can rule out citrullinemia type 2 (citrin deficiency). Since enzymatic analysis is complex [207], mutation identification is preferred and may help decide on the necessity of dietary therapy [27,78,206], since certain genotypes are associated with a mild phenotype [27]. The report of fatal hyperammonemia under severe catabolic circumstances in some of these asymptomatic patients [23,78,208,209] highlights the need for appropriate monitoring and for adopting the emergency protocol for hyperammonemia.

#### Statement #46. Grade of recommendation: C

Although in citrullinemia type 1 genetic analysis is the method of choice for diagnostic confirmation and for prenatal testing, it can reveal mild variants of citrullinemia type 1 that might need no therapy but are still at risk of metabolic decompensation. Enzyme analysis using an indirect assay in cultured fibroblasts can also confirm the diagnosis.

### ASL deficiency (argininosuccinic aciduria)

High plasma and urine ASA levels are pathognomonic of ASLD. Mutation identification facilitates prenatal testing (amniotic fluid ASA level can also be used, see Table 3) and can guide prognosis by associating specific mutations with variant clinical courses [15,50,210,211], whereas enzyme assay, by giving the residual ASL level, can guide management [50]. If arginine is supplied, hyperammonemia risk is low in ASLD, since two waste nitrogen atoms are excreted with each ASA molecule. Despite good ammonia control, many ASLD patients experience unremitting intellectual decline, suggesting brain toxicity by ASA [130,212], decreased amounts of guanidinoacetate [213], arginine therapy or alterations in NO production [214]. Thus, dosages of L-arginine exceeding those in other UCDs are not recommended, with a target plasma arginine level < 200 μmol/L (generally considered a safe level, [108]). Some centers reduce protein intake when plasma ASA level is >400 μmol/L, but the benefit of this approach remains to be proven. Neonatal (and apparently also late-onset) ASLD patients can present with hepatomegaly and progressive liver disease [215], the latter apparently associated with elevated plasma triglycerides (C. Dionisi-Vici and J.V. Leonard, personal communications). For unknown reasons ASLD is occasionally associated with reduced serum potassium requiring oral K^+^ supplementation (C. Dionisi-Vici, personal communication).

#### Statement #47. Grade of recommendation: C

Neither genetic analysis nor enzyme activity determinations are needed to confirm the diagnosis of ASLD. Genetic analysis is recommended for prenatal testing but ASA determination in the amniotic fluid is also reliable.

### Statement #48. Grade of recommendation: D

ASLD treatment exclusively with high-dose L-arginine supplementation is not recommended because of possible side-effects. For long-term management L-arginine should be given at the same dosages as for other UCDs in combination with sodium benzoate and protein restriction.

### Arginase 1 deficiency (argininemia)

ARG1D markedly differs from other UCDs in that it rarely manifests in the neonatal period [216-218], causing progressive spastic paraplegia and developmental delay between 2 and 4 years of age, with hepatomegaly and in some cases with hyperammonemic episodes [21] although metabolic decompensations are rarer than in other UCDs [21,219]. The increased plasma arginine level is the disease hallmark, but this increase may be small [220], rendering highly desirable the confirmation of the diagnosis by enzymatic assays easily carried out in erythrocytes [76] or by genetic analysis [219,221]. Increased urinary orotic acid excretion also supports the diagnosis [222].

Since arginine and its metabolites (e.g. guanidinoacetate, also found elevated in ARG1D [21,223]) are likely to be toxic, the goal is to reduce plasma arginine below 200 μmol/L. This requires severe natural protein restriction [220] and increased use of EAA supplementation (up to ~50% of the protein requirement). Sodium benzoate and sodium phenylacetate [224] are used to support this treatment, which should ideally halt disease progression [21] although it is uncertain if it can reverse the spastic diplegia.

#### Statement #49. Grade of recommendation: D

ARG1D manifestations differ remarkably from those in other UCDs. Treatment follows the standard UCD recommendations (without the use of L-arginine) but requires particularly severe protein restriction to reduce plasma arginine levels to <200 μmol/L.

### HHH syndrome

The manifestations differ from those of other UCDs and almost always include spastic paraparesis, which sets in more slowly than in ARG1D, preceded by hyperreflexia and other pyramidal signs [22,225]. Liver dysfunction and coagulopathy, with defects of factors VII, IX and X, are more common than in other UCDs [35,225]. The characteristic biochemical profile comprising a combination of hyperammonemia, elevated plasma ornithine and urinary excretion of homocitrulline and frequently also of orotate [226], generally renders further confirmatory tests unnecessary (such as determination of ^14^C-ornithine incorporation into protein in cultured skin fibroblasts or liver tissue [227], or mutation analysis [22,228]). Long-term therapy, consisting of a low-protein diet and L-citrulline supplementation (Table 8) prevents hyperammonemia and liver disease but apparently not the spastic paraparesis [22,225,229] although further studies are needed to assess the impact of treatment on the neurological derangements. Creatine deficiency was reported in this syndrome [229,230], supporting creatine supplementation if plasma creatine levels are low.

#### Statement #50. Grade of recommendation: D

Low-protein diet, *L*-citrulline and maybe also creatine supplementation are recommended in the HHH syndrome. The impact of these measures on neurological outcome remains to be ascertained.

## New trends and emerging therapies

### Potential new approaches for diagnosis, prognosis and novel therapies

Whole body ureogenic flow, assessed by using heavy isotopes and mass spectrometry, was shown to detect poor CPS1 activation in NAGSD [132,135,231,232] and was potentially useful for measuring in vivo urea cycle function. Use of MRS to detect elevated glutamine and decreased myo-inositol brain levels was reported to identify symptomatic and asymptomatic subjects with partial OTCD [173,174]. Advances in genotyping and in mutation analysis techniques should increase the sensitivity of mutation identification [81,200,233]. The increasing number of “normal” human genomes sequenced [234] should facilitate the distinction between polymorphisms and disease-causing mutations. Ongoing advances in modelling algorithms combined with improved knowledge of protein structure allow increasingly accurate prediction of the consequences of point mutations [235,236], although structures still remain to be determined for ORNT1, NAGS and CPS1 (but the human CPS1 C-terminal domain structure is known, see [237]). Structural information may also open the way to disease- and mutation-tailored pharmacological approaches [237]. Improved expression systems (see for example [238]) can be the bases for screening the effects of novel agents on mutant proteins, as exemplified recently with phenylalanine hydroxylase for treating phenylketonuria [239]. In this respect, the range of animal models for UCDs is still limited and some do not accurately replicate the human deficiency as in the case of the mouse knockout model for ARG1D [240]. Therefore, the development of other models such as conditional knockouts or transgenic mutants is essential.

### Experimental therapies

* Neuroprotective measures * based on the use of pharmacological agents [241-245] or of hypothermia [246,247] are under investigation to treat hepatic and hyperammonemic encephalopathy. Mild systemic hypothermia (rectal temperature of 34°C for 48 h), together with hemofiltration in a neonatal UCD patient with hyperammonemic coma was reported [248], resulting in a striking fall in plasma ammonia. A randomized controlled study for the use of hypothermia in neonatal hyperammonemia is planned in the US (U. Lichter-Konecki, personal communication).

#### Statement #51. Grade of recommendation: D

Hypothermia might be a supportive therapeutic option in acute hyperammonemia that is under study. Its use cannot be recommended outside controlled studies.

* Hepatocyte transplantation * is being proposed [249-252] as a bridging therapeutic measure in compromised UCD patients while waiting for liver transplantation [251]. Currently, there are studies underway testing the safety and efficacy of the procedure.

#### Statement #52. Grade of recommendation: D

Hepatocyte transplantation is still an experimental technique that should only be used as a part of standardized study protocols.

* Gene transfer * studies have been carried out in animal models of OTCD and ASSD [253-258], with report of long-term OTCD correction by using woodchuck hepatitis virus post-transcriptional regulatory element (WPRE)-mediated overexpression and a helper-dependent adenovirus [253]. One adenoviral gene therapy study for OTCD carried out in humans did not efficiently increase enzyme activity but caused inflammation and death in one enrolled subject [259,260]. Therefore, the promise of * gene therapy * remains to be fulfilled for UCDs, although the encouraging results from animal studies support continued efforts in this area.

#### Statement #53. Grade of recommendation: D

At present, there is no role for gene therapy in the treatment of UCDs.

Since arginase catalyzes a hydrolytic reaction, ARG1D is the one UCD in which enzyme replacement therapy is most likely to work. Prior attempts have been based on the use of virus-encoded [261] or erythrocyte-contained arginase [262,263]. Frequent blood transfusions help to almost normalize plasma arginine [263] and might be useful for gaining time in order to start definitive therapy [262]. However, pegylated human recombinant arginase 1 (PEG-BCT-100) is now available and is being used in a clinical trial for cancer ( http://clinicaltrials.gov/ct2/show/NCT00988195), opening the way to potential testing of its efficacy in ARG1D.

#### Statement #54. Grade of recommendation: D

At present, there is no role for enzyme replacement therapy in the treatment of UCDs.

### Closing remarks

Although these guidelines are the result of a three-year Delphi process aiming at delivering the best available level of evidence for any given recommendation, the rarity of these diseases, with little clustering of cases in single centers, has resulted in mostly C or D levels of evidence for the statements made here, which correspond to inferences derived from non-analytical studies such as case reports or case series or from expert opinion. Therefore, the recommendations contained herein should not be considered infallible or absolute. The working group of these guidelines commits itself to revise the work in the future in an effort to preserve the achieved quality and to search for higher evidence levels that might accrue with time. Indeed, it is hoped that many of the statements will be substituted in forthcoming years by even more precise and effective recommendations to the benefit of the patients. Moreover, the impact of these guidelines on patients’ outcome is planned to be evaluated after some years which will in addition help to improve any updated guidelines, possibly increasing also the level of evidence.

## Competing interests

The authors of these guidelines declare no competing interests but disclose the following: JH has received financial support for attending conferences from Orphan Europe and Swedish Orphan and holds a contract with Orphan Europe for performing genetic studies in patients suspected for NAGSD or CPS1D. The UCH Heidelberg (ML) has received research support from Cytonet GmbH for liver cell transplantation studies. In addition ML has received financial support for attending conferences and/or giving lectures on different topics from Orphan Europe, Swedish Orphan, and Nutricia. MD has received financial support to attend conferences and for giving lectures/workshops on different IMD topics from Nutricia, Vitaflo and Orphan Europe. VV and CDV received travelling grants and speaker fees from Orphan Europe unrelated to this work. VR has received conference fees and financial support for attending conferences from Swedish Orphan.

## Authors’ contributions

All authors of this work have contributed to the underlying Delphi process, to the planning, writing and revising of this paper. All authors read and approved the final manuscript.
